# Within-session changes in therapist and client behaviors during an alcohol brief motivational intervention for young men

**DOI:** 10.1186/1940-0640-8-S1-A30

**Published:** 2013-09-04

**Authors:** Jacques Gaume, Molly Magill, Richard Longabaugh, Nicolas Bertholet, Gerhard Gmel, Jean-Bernard Daeppen

**Affiliations:** 1 Center for Alcohol and Addiction Studies, Brown University, Providence, RI, USA; 2 Alcohol Treatment Centre, Lausanne University Hospital, Lausanne, Switzerland

## 

Brief motivational intervention (BMI) has shown promising results among young adults, but its underlying mechanisms are seldom investigated. Analyzing the dynamic processes of therapist and client behaviors throughout the session might help to better understand mechanisms operative during BMI. We used data from a BMI randomized controlled trial for heavy drinking among non-treatment seeking Swiss young men. The parent study found significantly lowered drinking in the BMI group (N=179) compared to a control group receiving no intervention (N=182) 3 months later. In the present study, we conducted psycholinguistic coding of 174 BMI using the Motivational Interviewing (MI) Skill Code (MISC 2.1; Miller et al. 2008) and then divided the sessions in thirds to examine within-session processes across time. Alcohol outcome was dichotomized into a “changers” group (baseline to 3-month difference greater than the mean of the control group) and a “non-changers” group. We then tested for interactions between time (thirds) and outcome group in GEE models accounting for within-person correlations across repeated (time) measures. Interactions were not significant for therapist frequency of MI-consistent behaviors, percent of open questions, and ratio of reflections to questions, but were significant for the frequency of MI-inconsistent behaviors (MIIN) and the percent of therapist reflections that were complex. Regarding client change talk, interactions were significant only for commitment to change. Findings indicated that there were dynamic processes at play during our BMI which were related to better alcohol outcomes. Specifically, the presence of MIIN in the beginning of a BMI appeared to be related to poor outcomes while an increase in complex reflections was related to good outcomes. As in prior MI process research, commitment to change strength was related to outcomes, but here the difference came from non-changers increasing their commitment not to change rather than from changers increasing their commitment to change.

